# Increased Detection of Viruses in Children with Respiratory Tract Infection Using PCR

**DOI:** 10.3390/ijerph17020564

**Published:** 2020-01-15

**Authors:** Chien-Yu Lin, David Hwang, Nan-Chang Chiu, Li-Chuan Weng, Hsin-Fu Liu, Jung-Jung Mu, Chang-Pan Liu, Hsin Chi

**Affiliations:** 1 Department of Pediatrics, Hsinchu MacKay Memorial Hospital, Hsinchu 30071, Taiwan; mmhped.lin@gmail.com; 2 Department of Medicine, MacKay Medical College, New Taipei City 25160, Taiwan; ncc88@mmh.org.tw (N.-C.C.); cpliu@mmh.org.tw (C.-P.L.); 3 Department of Pediatrics, MacKay Children’s Hospital and MacKay Memorial Hospital, Taipei 10449, Taiwan; Ckdarpay@gmail.com; 4 Department of Medicine, MacKay Junior College of Medicine, Nursing and Management, Taipei 11260, Taiwan; 5 Department of Medical Research, MacKay Memorial Hospital, Tamshui, New Taipei City 25160, Taiwan; lichuan@mmh.org.tw (L.-C.W.); hsinfu@mmh.org.tw (H.-F.L.); 6 Centers for Research, Diagnostics and Vaccine Development, Centers for Disease Control, Taipei 10050, Taiwan; jjmu@cdc.gov.tw

**Keywords:** respiratory virus, polymerase chain reaction, PCR, respiratory syncytial virus, human metapneumovirus, multiplex quantitative real-time RT-PCR

## Abstract

Respiratory viruses are a common cause of respiratory tract infection (RTI), particularly in neonates and children. Rapid and accurate diagnosis of viral infections could improve clinical outcomes and reduce the use of antibiotics and treatment sessions. Advances in diagnostic technology contribute to the accurate detection of viruses. We performed a multiplex real-time polymerase chain reaction (PCR) to investigate the viral etiology in pediatric patients and compared the detection rates with those determined using traditional antigen tests and virus cultures. Fifteen respiratory viruses were included in our investigation: respiratory syncytial virus A/B (RSV), influenza virus A (FluA) and influenza virus B (FluB), human metapneumovirus (MPV), enterovirus (EV), human parainfluenza virus (PIV) types 1–4, human rhinovirus (RV), human coronavirus OC43, NL63, and 229E, human adenovirus (ADV), and human bocavirus (Boca). In total, 474 specimens were collected and tested. Respiratory viruses were detected more frequently by PCR (357, 75.3%) than they were by traditional tests (229, 49.3%). The leading pathogens were RSV (113, 23.8%), RV (72, 15.2%), PIV3 (53, 11.2%), FluA (51, 10.8%), and ADV (48, 10.1%). For children younger than 5 years, RSV and RV were most prevalent; for children older than 5 years, FluA and ADV were the most frequently detected. Of the specimens, 25.8% (92/357) were coinfected with two or more viruses. RV, Boca, PIV2, FluB, and PIV4 had higher rates of coinfection; MPV and PIV1 had the lowest rates of coinfection (9.1% and 5.3%). To conclude, the detection power of PCR was better than that of traditional antigen tests and virus cultures when considering the detection of respiratory viruses. RSV and RV were the leading viral pathogens identified in the respiratory specimens. One-quarter of the positive specimens were coinfected with two or more viruses. In the future, further application of PCR may contribute to the rapid and accurate diagnosis of respiratory viruses and could improve patient outcomes.

## 1. Introduction

Respiratory viruses are ubiquitous and cause a large variety of clinical symptoms. Respiratory tract infection (RTI) is undoubtedly common, and the recognition of a causative pathogen contributes to the appropriate management [1]. In addition to the well-known respiratory viruses, such as respiratory syncytial virus (RSV) and influenza virus, human metapneumovirus (MPV) was identified in 2001, followed by the discovery of other respiratory viruses [2,3]. Currently, the disease burden of respiratory viruses is beyond our knowledge. Respiratory viruses have been detected in more than two-thirds of children with radiographically confirmed community-acquired pneumonia (CAP) [4]. Similarly, in the United States, molecular diagnostics revealed viral infection in 43%–67% of pediatric CAP cases [5]. Respiratory viruses also play an important role in adult pneumonia and are detected in 15%–56% of adult CAP cases [5,6]. Viruses are responsible for the majority of respiratory infectious diseases in both children and adults, causing a massive disease burden [7,8]. Furthermore, the identification of causative viruses enables the accurate diagnosis of respiratory infections and prescription of specific antiviral agents against certain viruses, such as oseltamivir for influenza viruses, and improves evaluation of the prognosis [9,10,11]. Recognizing causative viruses can also provide information on the appropriate infection control measures, which can potentially reduce unnecessary hospital stays and allow discontinuation of unnecessary antibiotics [12,13,14]. In summary, respiratory virus infection is common, and testing for respiratory pathogens can improve understanding of the roles of pathogens in respiratory diseases and contribute to their better clinical management [15].

A timely and accurate diagnosis of viral infection can be challenging. Rapid antigen tests are used to detect influenza virus infection worldwide, but there are some concerns regarding the sensitivity of currently available viral antigen tests [6,15]. Technological advances have improved the sensitivity, accessibility, and utility of viral diagnostic tools [16]. Molecular assays have been developed and progressively multiplexed to diagnose a large number of respiratory viruses in a single assay with excellent sensitivity and specificity [10,17,18,19,20]. The importance of molecular-based diagnostic modalities is currently on the rise, and polymerase chain reaction (PCR) technology is being increasingly used in the clinic to rapidly diagnose respiratory infections [19]. This study aims to detect respiratory viruses in children using PCR and to compare the detection power of this technique against that when using traditional antigen tests and virus cultures. The clinical conditions were also investigated.

## 2. Materials and Methods

### 2.1. Study Design and Sample Collection

This study was approved by the Institutional Review Board of the MacKay Memorial Hospital, Taipei, Taiwan (approval no. 14MMHIS030). For children with respiratory symptoms and with a clinical suspicion of virus infection, a test for RSV antigen test, human parainfluenza virus (PIV) type 3 antigen test, viral PCR for enterovirus, or viral cultures was prescribed by the judgment of pediatricians. A nasopharyngeal swab or aspiration was performed by pediatricians using a small swab that was inserted into the nostril. The cotton swab was then inserted and mixed in a 2.5 mL viral transport medium. After testing original tests, the residual specimens were stored at −20 °C in the clinical viral laboratory at the Department of Laboratory Medicine, Mackay Memorial Hospital. Within a week, the samples were transported to a deep freezer (−70 °C) at the Department of Medical Research, Mackay Memorial Hospital, for present multiplex PCR tests. Commercialized antigen diagnostic kits were used for antigen tests (QuickVue assay, Quidel Corporation, San Diego, CA, USA) and tube cultures were used for virus cultures. The cell lines for virus cultures were medical research council cell line strain 5 (MRC-5, ATCC^®^ CCL-171^TM^), human epithelial cell line type 2 (HEp-2, ATCC^®^ CCL-23^TM^), human adenocarcinomic alveolar basal epithelial cell line (A549, ATCC^®^ CCL-185^TM^), Madin–Darby canine kidney cell line (MDCK, ATCC^®^ CCL-34^TM^), and human rhabdomyosarcoma cell line (RD, ATCC^®^ CCL-136^TM^). Additionally, viruses commonly isolated were adenovirus, MPV, RSV, influenza virus type A (FluA) and B (FluB), parainfluenza virus types 1, 2, and 3; echovirus 4, 6, 9, 11, and 30; coxsackievirus B1–B6; enterovirus 70; enterovirus 71; pan-enterovirus; herpes simplex virus types 1 and 2; cytomegalovirus; coxsackievirus A9, A16, and A24. The residual specimens were stored, and then present multiplex PCR was performed for RTI viruses.

### 2.2. Extraction of Viral Nucleic Acids, Reverse Transcription, and Multiplex RT-PCR Analysis

The viral nucleic acids were extracted from 200-µL of each sample using the High Pure Viral Nucleic Acid Kit (Roche Applied Science, Castle Hill, Germany) following the manufacturer’s instructions. Extracted nucleic acids were eluted in 100 μL elution buffer and stored at −70 °C. Reverse transcription (RT) was carried out using High-Capacity Complementary DNA (cDNA) Reverse Transcription Kit (Applied Biosystems Part Number: 4375575 Rev.C). The total volume of RT mix was 40 μL per reaction, containing 4 μL RT buffer (10×), 1.6 μL dNTP mixture (25 mM of each dNTP), 4 μL random primers (10×), 2 μL RNase inhibitor (20 U/μL), 2 μL MultiScribe Reverse Transcriptase (50 U/μL), and 26.4 μL template, whereby the RT reagent mix was prepared on ice. The thermal profile of the RT program consisted of 10 min incubation at 25 °C, 120 min RT at 37 °C, 5 min RT inactivation at 85 °C, and cooling down to 4 °C and was performed in a 96-well GeneAmp PCR System 9700. The resulting cDNA was stored at −20 °C.

The following multiplex PCR assays were performed for each sample to detect RNA/DNA of 15 respiratory viruses, including RSV A or B, FluA, FluB, human enterovirus (EV), MPV, human parainfluenza virus types 1–4, human rhinovirus (RV), coronavirus OC43/NL63/229E, human adenovirus (ADV), and human bocavirus (Boca). In the present study, previously published primers and PCR assays were used for multiplex RT-PCR and the details of primers are summarized in Table S1 [21,22,23,24,25,26]. Briefly, the PCR reaction was performed by adding 3 µL RT product to 22 µL PCR mix. The conditions of amplification were as follows: initial denaturation at 95 °C for 10 min; followed by 40 cycles of 95 °C for 1 min, 60 °C for 1 min, and 72 °C for 1 min; a final extension at 72 °C for 10 min. Amplification products were visualized by 1% agarose gel electrophoresis with ethidium bromide staining and observed under ultraviolet light. For each PCR assay, a positive and negative control for each parameter was performed. Internal control was also performed to detect sample inhibition and avoid false-negative results. External and internal amplification controls were designed for quality control and validation. The detection limits of the multiplex PCR assays were 10 to 100 copies of the individual virus.

### 2.3. Statistical Analysis

Student’s *t*-test and chi-square test were used to analyze and compare the categorical demographic characteristics including clinical manifestations and laboratory tests. Kappa statistic was used to evaluate the consistency between PCR and original tests (categorical variables) and Cohen’s kappa coefficient (*κ*) was regarded as poor to fair consistency if *κ* ≤ 0.4; moderate consistency if 0.41 ≤ *κ* ≤ 0.60; and good consistency if 0.61 < *κ*. A two-sided *p* < 0.05 was considered statistically significant. Statistical analyses were performed using the SPSS software version 23.0 (SPSS Inc., Chicago, IL, USA).

## 3. Results

**Figure 1:**
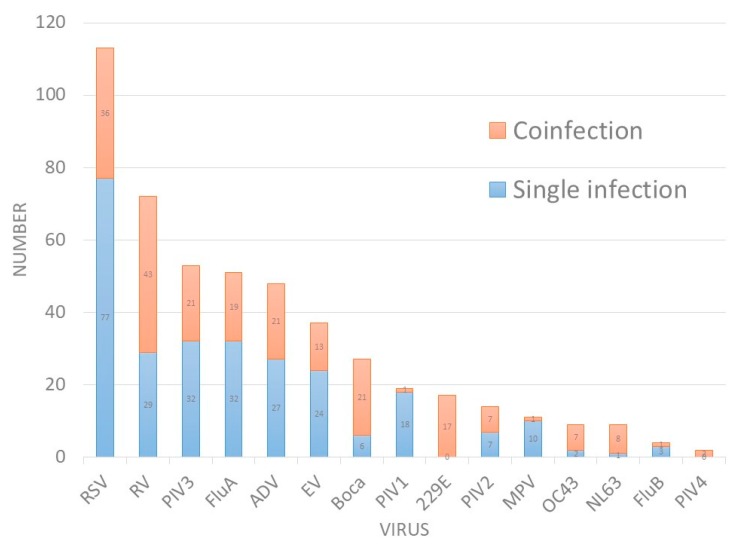
Number of respiratory viruses detected by PCR.

In total, 474 residual specimens for detecting respiratory viruses were obtained, including 156 specimens for RSV antigen tests, 58 for parainfluenza virus antigen tests, and 260 for viral cultures. Table 1 summarizes the detection rates of viruses. The overall positive rate for traditional tests was 48.3% (229/474), and the individual positive rate was 28.8% for RSV antigen tests, 5.2% for parainfluenza virus antigen tests, and 69.6% for viral cultures. All specimens underwent present multiplex PCR for the 15 abovementioned viruses, and higher detection rates were observed; 357 (75.3%) specimens were positive for at least one virus. The leading pathogens were RSV (113, 23.8%), RV (72, 15.2%), PIV3 (53, 11.2%), FluA (51, 10.8%), and ADV (48, 10.1%) (Figure 1). Among these positive specimens, 25.8% (92/357) were coinfected with two or more viruses. The coinfection rates of individual virus were demonstrated in Table 1. We observed that RV, Boca, PIV2, FluB, and PIV4 were associated with higher rates of coinfection. However, MPV and PIV1 had the lowest rates of coinfection (9.1% and 5.3%). The consistency of the results between virus culture and PCR was also investigated. With the exception of FluB, a high consistency was observed between virus culture and PCR (coefficient *k*: 0.72~0.961, *p* < 0.01, Table 1).

**Figure 2:**
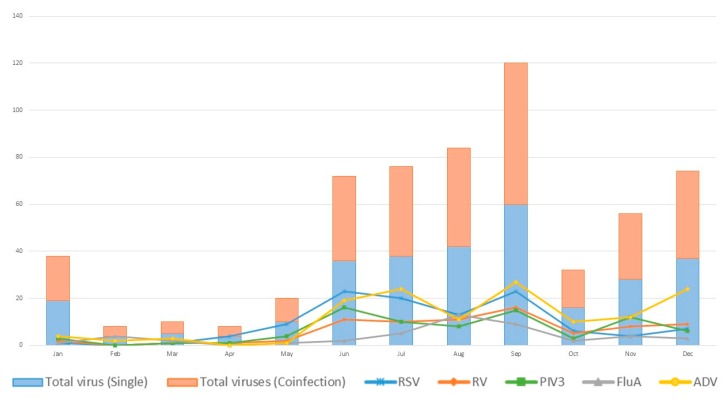
Seasonal distribution of respiratory viruses detected by PCR. (Abbreviations: 229E: coronavirus 229E; ADV: human adenovirus; Boca: human bocavirus; EV: human enterovirus; Flu: influenza virus; MPV: human metapneumovirus; NL63: coronavirus NL63; OC43: coronavirus OC43; PIV: human parainfluenza virus; RSV: respiratory syncytial virus; RV: human rhinovirus).

**Figure 3:**
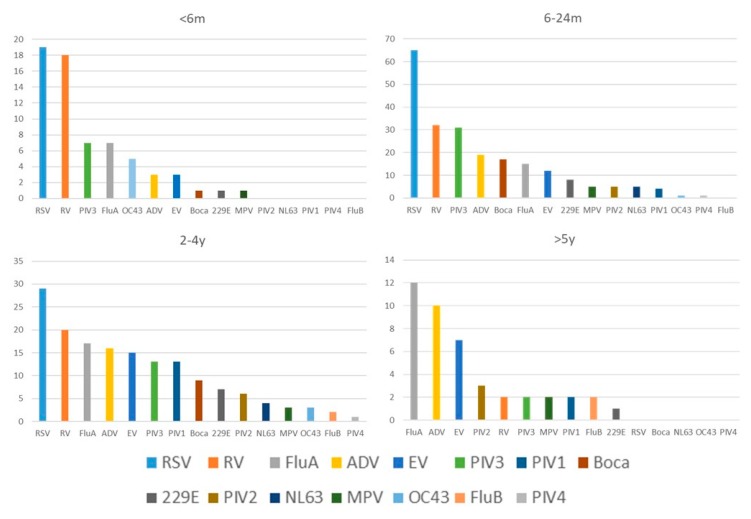
The distribution of respiratory viruses among different age groups. (Abbreviations: 229E: coronavirus 229E; ADV: human adenovirus; Boca: human bocavirus; EV: human enterovirus; Flu: influenza virus; MPV: human metapneumovirus; NL63: coronavirus NL63; OC43: coronavirus OC43; PIV: human parainfluenza virus; RSV: respiratory syncytial virus; RV: human rhinovirus).

The seasonality of virus detection is shown in Figure 2; virus detection was more common in summer and autumn. The seasonal distribution of the five most commonly detected viruses (RSV, RV, PIV3, FluA, and ADV) was also plotted. We also compared the detection rate in different age groups (Figure 3). For children younger than 5 years, RSV and RV were the leading pathogens; for older children, FluA and ADV were prevalent. The clinical manifestations and laboratory tests are summarized in Table 2 (complete data available in Table S2). Except for age, no obvious differences were found between individual viruses. More than one-quarter of the specimens were coinfected with more than one virus. We further compared the clinical manifestations of patients in which either no viruses, a single infection, or coinfections were detected. The age, body weight, duration of hospitalization, intensive care unit (ICU) stay, white blood cell counts (WBC), and C-reactive protein (CRP) levels were not significantly different, with higher platelet counts being the only difference noted in patients with coinfections (Table 3).

## 4. Discussion

In this study, we found that PCR had higher detection rates compared with traditional antigen tests and viral cultures (75.3% vs. 48.3%). RSV, RV, and PIV3 were the leading pathogens detected in pediatric RTI patients. However, FluA, ADV, and EV were more prevalent in children older than 5 years. Knowledge of epidemiology contributes to the awareness of pathogen, accurate diagnosis, and prompt management. We also found that approximately one-quarter of specimens were coinfected with two or more viruses. However, no obvious differences in clinical manifestations and laboratory tests were found in individual virus infection or between single infection and coinfection; the clinical significance of coinfection was not fully elucidated.

A rapid and accurate diagnosis of respiratory viruses is increasingly important in clinical settings. The availability of rapid diagnostic assays is essential for optimizing the efforts of infection control teams to reduce the transmission of virulent or resistant pathogens in hospitals [27]. Nucleic acid amplification tests are the new gold standard for the diagnosis of respiratory viruses. Our study has shown high detectability of PCR for respiratory viruses, suggesting that PCR-based diagnostic tools may be practical for detecting a wide range of respiratory viruses. Viral infection can be fatal, especially in premature infants and infants with congenital heart disease [28]. In a previous study, symptomatic and asymptomatic premature infants were prospectively screened in a neonatal ICU using multiplex PCR twice weekly; respiratory viruses were identified in 52% of prematurely born infants during their birth hospitalization. Their length of hospital stay was significantly longer (70 days vs. 35 days), and bronchopulmonary diseases were more frequent in infected infants [8]. In adult and pediatric patients, the major impact of respiratory viral infections with hematologic malignancies, hematopoietic stem cell transplantation, and solid organ transplantation has been recognized over the past decade [7,28]. In the most immunocompromised populations, respiratory viruses have a high rate of progression to pneumonia (20%–40%). The mortality among those patients ranged from 30% to 50%. The application of multiplex PCR for respiratory virus detection in high-risk groups has been proved to be valuable [17]. Our study showed a high detectability of PCR for respiratory viruses, suggesting that PCR-based diagnostic tools may be helpful for detecting a wider range of respiratory viruses. We also showed a high consistency of PCR with virus cultures, except for FluB, suggesting the accuracy of the PCR method. Virus culture is time-consuming and not feasible for clinical practice. It was even impossible to detect some viruses by virus cultures, e.g., coronaviruses (229E, OC43, NL63, and HKU-1), PIV4, RV, and Boca. Approximately just over half of the viruses could be detected after the wide application of PCR. These results re-enforce the importance of PCR-based diagnosis.

Viral infections are ubiquitous and may present with fever and respiratory symptoms. It is sometimes difficult to differentiate between bacterial infections and viral infections, and thus the use of unnecessary antibiotics is common. Antimicrobial resistance (AMR) has been increasing worldwide, resulting in poor treatment responses and deplorable clinical outcomes [29]. The problem of AMR is an urgent and critical health threat and is directly associated with the overuse of antibiotics [30]. Antibiotic treatment does not improve the clinical outcomes of viral infections [31]. Decreasing the use of unnecessary antibiotics is the key to combating AMR, and accurate and rapid diagnosis is crucial to decrease antibiotic prescriptions with a minimized risk [9,10,32]. The present study demonstrates that PCR has higher detectability for respiratory viruses compared to traditional antigen tests and viral cultures. PCR-based viral detection may help physicians to make appropriate decisions and decrease unnecessary antibiotic use. Furthermore, the precise diagnosis of certain viruses may contribute to timely antiviral agent treatment, e.g., oseltamivir against influenza infections. We discovered that influenza is common among pediatric patients (11.6% of respiratory specimens) and is the most commonly detected pathogen in older children (27% in children aged 5–9 years and 16.7% in children older than 10 years). Rapid diagnosis of influenza viruses and early treatment with oseltamivir or peramivir is crucial. In addition, prompt diagnosis of respiratory viruses also contributes to appropriate infection control measures and isolation care [8,27]. In recent years, the cost of PCR testing has decreased, and the availability and feasibility has been largely improved. Some commercialized PCR machines are increasingly available and may serve in point-of-care testing [18]. Hence, the widespread use of PCR-based detection of respiratory viruses is increasing and may become more practical.

The incidence of etiologic pathogens differs between adults and children. It has been reported by Jain et al. that, among the hospitalized adults with CAP, pathogens were detected in 38% of patients, and the leading pathogens were RV (9%) and influenza viruses (6%) [6]. By contrast, pathogens were detected in 81% of the hospitalized CAP children, and the leading pathogens were RSV (28%), RV (27%), and MPV (13%) [4]. Generally speaking, viral infections are more prevalent in children than in adults. The leading pathogens may also differ according to geographical region, climate, season, and year. The leading pathogens detected in our study were RSV (23.8%), RV (15.2%), and PIV3 (11.2%). A previous study conducted in Taiwan found that RSV was the most common pathogen (41.7%), followed by MPV (27.1%), Boca (6.3%), and EV (6.3%) [33]. Some important studies investigating the epidemiology of respiratory tract infection are summarized in Table 4 [4,6,13,15,23,34,35]. RSV is always the most common pathogen in young children worldwide, but the accompanying pathogens are not always the same [4,5,7,13,36,37]. Virus detection was more common in summer and autumn in our study. Taiwan is located in a subtropical zone, where there are no swift changes in temperature amplitudes. Although RSV infections occur biennially, with peaks reported in the spring and autumn in Taiwan, variations in RSV infections are not particularly large [38,39,40]. Detection of respiratory viruses could enable estimation of the local epidemiology of respiratory viral infection and help pediatricians to improve their clinical judgments.

One-quarter of the positive specimens were coinfected with other respiratory viruses in our study. A similar prevalence was found in previous studies [4,41,42]. The rates of coinfection were between 18.8% to 36.2% in previous studies (Table 4). With the advances in diagnostic testing, the number of detectable viruses will increase. However, the clinical significance of coinfection remains unclear [43,44,45,46]. Some studies reported increased severity of coinfection [45,46], but the impact of coinfection was not particularly obvious in other studies [47,48]. Diversities in the study design, population, and detection methods may be the reason for this inconclusiveness. When we compared the clinical manifestations and laboratory tests for patients with negative detection, single infection, and coinfections, we found no statistically significant differences in age, body weight, hospitalization duration, ICU stay, CRP level, and complete blood cell counts; although higher platelet counts were observed in patients with coinfection. Further studies are required to clarify the clinical significance of our findings.

The strength of our study lies in the comprehensive detection of respiratory viruses and further comparison of the clinical manifestations and laboratory tests in single and coinfection. Our study is subject to some limitations that warrant discussion. Firstly, although our findings were consistent with those of previous studies, respiratory specimens were not collected in all patients with respiratory symptoms. The prevalence of EV was underestimated because the clinical diagnosis of EV infection relies mainly on the presence of oral vesicles. Further virus culture might not be performed when vesicles over oropharynx were found. Secondly, we did not include bacteria in our detection spectra. Some bacteria such as *Mycoplasma pneumoniae* and *Streptococcus pneumoniae* also play an important role in respiratory infections and commonly cause coinfections with other pathogens [49]. Furthermore, some respiratory viruses were not included in our testing, such as the Middle East respiratory syndrome coronavirus and human polyomaviruses KI and WU [50].

## 5. Conclusions

The use of PCR resulted in greater detection of respiratory viruses than the use of traditional rapid antigen tests or viral cultures. More than half of the respiratory specimens that showed negative detection in the original tests were positive for the PCR-based detection method. Further application of PCR has great potential for rapid and accurate diagnosis and will be beneficial for primary pediatricians. Furthermore, RSV and RV were the leading pathogens identified in our pediatric respiratory specimens; in children older than 5 years, FluA, ADV, and EV were more prevalent. Approximately one-quarter of the positive respiratory specimens were coinfected with two or more viruses, but no obvious differences in clinical manifestations and laboratory tests were observed between single infection and coinfection. Further studies are warranted to investigate the accuracy, feasibility, accessibility, and cost of PCR in detecting respiratory viruses, and to clarify the clinical significance of coinfection.

## Figures and Tables

**Table 1:** Detection rates of individual viruses using different methods.

Viruses	PCR			Traditional Tests		Coefficient *k*	*p* for *k*
	Positive No (%)	Coinfection	Coinfection Rates	Positive No (%)	Coinfection		
Total	357 (75.3)	92	25.8%	229 (48.3)	30		
RSV	113 (23.8)	36	31.9%	70 (9.6)	4	0.72	<0.01
RV	72 (15.2)	43	59.7%	-	-		
PIV3	53 (11.2)	21	39.6%	21 (6.9)	3	0.77	<0.01
FluA	51 (10.8)	19	37.3%	47 (18.1)	5	0.92	<0.01
ADV	48 (10.1)	21	43.8%	30 (11.5)	9	0.8	<0.01
EV	37 (7.8)	13	35.1%	29 (11.2)	7	0.87	<0.01
Boca	27 (5.7)	21	77.8%	-	-		
PIV1	19 (4)	1	5.3%	13 (5.0)	3	0.925	<0.01
229E	17 (3.6)	17	100.0%	-	-		
PIV2	14 (3.0)	7	50.0%	13 (5.0)	3	0.961	<0.01
MPV	11 (2.3)	1	9.1%	-	-		
OC43	9 (1.9)	7	77.8%	-	-		
NL63	9 (1.9)	8	88.9%	-	-		
FluB	4 (0.8)	1	25.0%	12 (4.6)	5	0.49	<0.01
PIV4	2 (0.4)	2	100.0%	-	-		

Abbreviations: ADV: adenovirus; Boca: human bocavirus; EV: enterovirus; Flu: influenza virus; MPV: human metapneumovirus; PCR: polymerase chain reaction; PIV: parainfluenza virus; RSV: respiratory syncytial virus; RV: human rhinovirus.

**Table 2:** Comparison of clinical characteristics of different viruses.

Viruses	Age (m/o)	BW (kg)	Hospital Days (day)	ICU	Hb	Hct	Plt	WBC	Neut (%)	CRP (mg/dL)
RSV	16.63	9.72	5.85	5	11.85	35.57	336,439	10,812	43	1.47
RV	19.25	10.21	5.93	4	11.96	35.88	350,282	12,949	50	1.66
PIV3	21.4	10.75	6.96	0	11.83	35.5	311,000	10,492	46.8	2.1
FluA	38.98	14.27	4.88	0	11.84	35.25	261,645	9156	54.78	2.59
ADV	39	15.02	6.46	2	11.68	35.1	298,575	13,415	57.84	4.65
EV	35.16	14.33	4.22	0	11.93	34.63	260,028	11,689	61.6	3.19
Boca	19.41	10	5.22	1	12.27	36.91	309,154	10,198	47.2	2.39
PIV1	34.42	13.94	6.74	0	12.17	36.44	233,737	7900	54.17	1.37
229E	27.53	12.1	4.41	1	12.2	36.03	323,765	13,912	54.6	2.29
PIV2	48.7	17.08	5.14	0	11.8	35.39	243,500	9350	57	2.07
MPV	26.55	12.05	6.55	0	12.03	35.75	255,455	7427	46.1	2.03
OC43	13.78	9.96	9.56	1	11.56	34.18	321,889	10,533	41.9	1.19
NL63	21.56	11.72	3.89	0	12	36	319,333	14,800	65.78	2.81
FluB	86	27.2	4.5	0	12.83	37.98	233,250	7800	74	1.985
PIV4	21.5	9.7	8	0	12.35	38.05	21900	8000	49.5	1.13

Abbreviations: ADV: adenovirus; ANC: absolute neutrophil count; Boca: human bocavirus; BW: body weight; CRP: C-reactive protein; EV: enterovirus; Flu: influenza virus; Hb: hemoglobin; Hct: hematocrit; ICU: intensive care unit; Lym: lymphocyte; MPV: human metapneumovirus; PCR: polymerase chain reaction; PIV: parainfluenza virus; Plt: platelet count; RSV: respiratory syncytial virus; RV: human rhinovirus; WBC: white blood cell count.

**Table 3:** Comparison of clinical characteristics of single pathogen and coinfection.

Variables	Coinfection (*N* = 92)	Single Pathogen (*N* = 265)	Negative (*N* = 117)	*p1*	*p2*
Age (m/o)	29.03 ± 34.2	27.06 ± 27.8	29.22 ± 35.19	0.582	0.617
BW (kg)	13.0 ± 10.3	12.2 ± 6.7	12.4 ± 8.8	0.419	0.971
Hospital days (day)	5.6 ± 4.06	5.7 ± 4.99	7.7 ± 11.56	0.852	0.063
ICU	2	10	5	0.924	-
ICU days	8.7 ± 14.2	3.5 ± 5	0	0.498	-
Hb	11.73 ± 1.82	11.78 ± 1.82	11.53 ± 3.27	0.841	0.454
Hct	34.75 ± 6.4	34.63 ± 7.18	34.44 ± 9.89	0.886	0.825
Plt	308,141 ± 133,332	286,935 ± 118,557	251,730 ± 117,792	0.154	0.002
WBC	10,693 ± 5499	10,746 ± 6039	10,436 ± 6137	0.942	0.643
ANC	5676 ± 4612	5802 ± 4803	5490 ± 4594	0.828	0.579
Band (%)	0.55	0.74	0.97	0.356	0.297
Neut (%)	49.5	50.7	47.7	0.648	0.27
Eos (%)	1.13	0.98	1.36	0.474	0.132
Baso (%)	0.27	0.17	0.18	0.08	0.693
Mono (%)	10.04	9.91	10.2	0.834	0.692
Lym (%)	36.78	34.7	34	0.394	0.56
Atyp Lym (%)	0.63	0.81	0.97	0.304	0.237
CRP (mg/dL)	2.03 ± 3.3	2.02 ± 3.25	2.59 ± 4.66	0.981	0.227

*p1*: *p*-value between single infection and coinfection; *p2*: *p*-value between coinfection and negative detection; Abbreviations: ANC: absolute neutrophil count; BW: body weight; CRP: C-reactive protein; Hb: hemoglobin; Hct: hematocrit; ICU: intensive care unit; Plt: platelet count; WBC: white blood cell count.

**Table 4:** Respiratory viruses detected by PCR in different countries.

Study	Country	Study Period	Study Population	Patient No.	Diagnosis	Positive Rate for Virus	1st Detected Virus	2nd Detected Virus	3rd Detected Virus	Coinfection Rate
Preeti 2009 [34]	India	2005–2007	Children	301	Lower RTI	35.2%	RSV 20.3%	PIV3 7.3%	PIV2 5.6%	18.8%
Childlow 2009 [23]	Australia	Jul 2006–Sep 2006	Children	121	RTI	71%	RSV 33.9%	RV 30.6%	ADV 8.3%	34%
Huijskens 2012 [15]	Netherland	Jan 2010–Dec 2010	Children	177	RTI	73%	RSV 36.6%	RV 24%	EV 8.5%	36.2%
Garcia-Garcia 2012 [13]	Spain	Sep 2004–Jul 2010	Children	884	CAP	73.4%	RSV 41.6%	RV 26.2%	Boca/ ADV 17.8%	30%
Jain 2015 [4]	USA	Jan 2010–Jun 2012	Children	2012	CAP	66%	RSV 28%	RV 27%	MPV 13%	26%
Jain 2015 [6]	USA	Jan 2010–Jun 2012	Adults	2320	CAP	23%	RV 9%	Flu A or B 6%	MPV 4%	8.3%
Jiang 2017 [35]	China	Jan 2015–Dec 2015	Children	846	CAP	70.1%	RSV 22.9%	RV 22.1%	Boca 6%	34.6%
Present study	Taiwan	Aug 2012–Jul 2014	Children	474	RTI	75.3%	RSV23.8%	RV15.2%	PIV3 11.2%	25.8%

Abbreviations: ADV: adenovirus; Boca: human bocavirus; CAP: community acquired pneumonia; EV: enterovirus; Flu: influenza virus; MPV: human metapneumovirus; PIV: parainfluenza virus; RSV: respiratory syncytial virus; RTI: respiratory tract infection; RV: human rhinovirus.

## Supplementary Materials

The following are available online at https://www.mdpi.com/1660-4601/17/2/564/s1. Table S1: Primers and PCR assays for multiplex PCR. Table S2: Comparison of clinical characteristics of different viruses.
