# Supplementary material for: Increased Detection of Viruses in Children with Respiratory Tract Infection Using PCR
Source: Int J Environ Res Public Health. 2020 Jan 15;17(2):564. doi: 10.3390/ijerph17020564 (PMC7013517; doi:10.3390/ijerph17020564)
Supplement: Supplementary file 1 [file ijerph-17-00564-s001.pdf]

Table S1. Primers and PCR assays for multiplex PCR.

Multiplex Assay	Primer Name	Direction	Sequence (5′–3′)	Target Gene	Amplicon (size, bp)	Virus	Highest 10-fold Dilution Detection
**Multiplex 1**	FluA_F	sense	CCTTCTAACCGAGGTCGAAACG	Matrix protein	241	FluA	10^{-6}
	FluA_R	antisense	GCATTTTGGACAAAGCGTCTACG				
	FluB_F	sense	AGACACAATTGCCTACCTGCTTTC	Matrix protein	352	FluB	10^{-6}
	FluB_R	antisense	CTGAGCTTTCATGGCCTTCTGC				
	RSV_F	sense	CATGACTCTCCTGATTGTGGGATG	Nucleocapsid	271	RSV	10^{-7}
	RSV_R	antisense	CCTTCAACTCTACTGCCACCTC				
	MPV_F	sense	TGAAGTCAATGCGACTGTAGCAC	Matrix protein	371	MPV	NA
	MPV_R	antisense	ATGCCTTTGGGATTGTTCATGGTC				
**Multiplex 2**	PIV1_F	sense	ATGATTTCTGGAGATGTCCCGTAGG	HA-NA	300	PIV1	10^{-8}
	PIV1_R	antisense	TTCCTGTTGTCGTTGATGTCATAGG				
	PIV2_F	sense	CAATCAATCCTGCAGTCGGAAGC	HA-NA	386	PIV2	10^{-6}
	PIV2_R	antisense	AAAGCGATGCAGACCACCAAG				
	PIV3_F	sense	GACACAACAAATGTCGGATCTTAGG	HA-NA	230	PIV3	10^{-6}
	PIV3_R	antisense	ATACAGCCATCAACAGTCGTTGG				
	PIV4_F	sense	CTGAACGGTTGCATTCAGGT	Phosphoprotein	451	PIV4	10^{-6}
	PIV4_R	antisense	TTGCATCAAGAATGAGTCCT				
**Multiplex 3**	RV_F	sense	CCCACAGTAGACCTGGCAGATG	5'-noncoding region	254	RV	NA
	RV_R	antisense	ACGGACACCCAAAGTAGTTGGT				
	CoV-229E_F	sense	GGTTTTGACAAGCCTCAGGAAAAAGA	Membrane glycoprotein	573	CoV-229E	10^{-8}
	CoV-229E_R	antisense	GTGACTATCAAACAGCATAGCAGCTGT				
	CoV-OC43_F	sense	GCTAGTCTTGTTCTGGCAAAACTTGGC	Membrane glycoprotein	335	CoV-OC43	10^{-8}
	CoV-OC43_R	antisense	TGAATTGCGCTATAACGGCGC				
**Multiplex 4**	ADV_F	sense	CAAAGCTCCCTAGGAAACGACCT		193	ADV	10^{-4}
	ADV_R	antisense	GCGGGTATGGGGTAAAGCATGT	Hexon			
	Boca_F	sense	GACCTCTGTAAGTACTATTAC		354	Boca	10^{-7}
	Boca_R	antisense	CTCTGTGTTGACTGAATACAG	Nonstructural protein-1			

Abbreviations: ADV: human adenovirus; Boca: human bocavirus; CoV: human coronavirus; EV: enterovirus; F: forward; FluA: influenza virus A; FluB: influenza virus B; RSV: respiratory syncytial virus; MPV: human metapneumovirus; PCR: polymerase chain reaction; PIV: human parainfluenza virus; R: reverse ; RV: human rhinovirus.

Table S2. Comparison of clinical characteristics of different viruses.

Viruses	RSV	RV	PIV3	FluA	ADV	EV	Boca	PIV1	229E	PIV2	MPV	OC43	NL63	FluB	PIV4
**Age (m/o)**	16.63 ± 12.34	19.25 ± 18.91	21.4 ± 24.8	38.98 ± 39.34	39 ± 31.9	35.16 ± 28.98	19.41 ± 11	34.42 ± 18.47	27.53 ± 21.45	48.7 ± 47.5	26.55 ± 21.14	13.78 ± 12.66	21.56 ± 13.62	86 ± 45.2	21.5 ± 4.5
**BW (kg)**	9.72 ± 2.99	10.21 ± 4.9	10.75 ± 5.43	14.27 ± 8.32	15.02 ± 8.76	14.33 ± 8.21	10 ± 2.61	13.94 ± 3.47	12.1 ± 4.43	17.08 ± 10.44	12.05 ± 4.33	9.96 ± 4.03	11.72 ± 2.33	27.2 ± 13.1	9.7 ± 0.3
**Hospital days (day)**	5.85 ± 3.98	5.93 ± 6.45	6.96 ± 4.82	4.88 ± 1.69	6.46 ± 6.64	4.22 ± 1.74	5.22 ± 3.17	6.74 ± 9.6	4.41 ± 1.78	5.14 ± 1.46	6.55 ± 4.27	9.56 ± 13.5	3.89 ± 1.29	4.5 ± 1.66	8 ± 1
**ICU**	5	4	0	0	2	0	1	0	1	0	0	1	0	0	0
**ICU days**	4.4 ± 3.44	3.25 ± 3.9	-	-	5.5	-	6	-	1	-	-	1	-	-	-
**Hb**	17.85 ± 1.07	11.96 ± 1.4	11.83 ± 1.19	11.84 ± 1.26	11.68 ± 1.14	11.93 ± 0.95	12.27 ± 1.51	12.17 ± 0.96	12.2 ± 0.92	11.8 ± 1.17	12.03 ± 1.03	11.56 ± 2.14	12 ± 0.74	12.83 ± 1.35	12.35 ± 0.25
**Hct**	35.57 ± 3	35.88 ± 4.2	35.5 ± 3.2	35.25 ± 3.64	35.1 ± 3.19	34.63 ± 5.86	36.91 ± 3.97	36.44 ± 2.89	36.03 ± 2.7	35.39 ± 3.27	35.75 ± 3.36	34.18 ± 6.57	36 ± 2.1	37.98 ± 4.12	38.05 ± 1.25
**Plt**	336,439 ± 124,267	350,282 ± 29,691	311,000 ± 13,937	261,645 ± 100,203	298,575 ± 106,730	260,028 ± 11,864	309,154 ± 10,6427	233,737 ± 62,686	323,765 ± 77,850	243,500 ± 62,857	255,455 ± 95,768	321,889 ± 115,965	319,333 ± 101,366	233,250 ± 35,759	21,900 ± 48,000
**WBC**	10,812 ± 5706	12,949 ± 5910	10,492 ± 6071	9156 ± 4883	13,415 ± 5617	11,689 ± 6406	10,198 ± 3709	7900 ± 3837	13,912 ± 5209	9350 ± 3758	7427 ± 3768	10,533 ± 2837	14,800 ± 5257	7800 ± 3124	8000 ± 3400
**ANC**	4846 ± 4114	6943 ± 5779	5381 ± 4816	5125 ± 3613	7899 ± 4614	7781 ± 6031	4958 ± 2853	4294 ± 2955	7758 ± 4422	5537 ± 3046	3563 ± 1935	4318 ± 2431	10216 ± 5567	5832 ± 2433	3943 ± 1643
**Band (%)**	0.46	0.38	0.66 ± 1.26	1.36	0.55	0.78	0.69	0.89	0.18	0.14	1.45	0.78	0.44	2.25	0
**Neut (%)**	43	50	46.8 ± 20.6	54.78	57.84	61.6	47.2	54.17	54.6	57	46.1	41.9	65.78	74	49.5
**Eos (%)**	0.87	2.01	1.38	0.61	0.83	0.72	1.23	0.52	0.98	0.68	1	3.22	1.11	0.5	0
**Baso (%)**	0.22	0.25	0.21	0.09	0.28	0.16	0.28	0.22	0.29	0.01	0.36	0.13	0.14	0	0.5
**Mono (%)**	10.6	9.84	10.1	10.85	9.87	9.06	9.66	9.27	11.14	9.72	9.73	8.87	7.66	7.75	13
**Lym (%)**	43.9	37.08	39.8	31.64	28.59	26.99	40.19	34.3	31.3	31.9	40.18	44.43	24.42	15	37
**Atyp Lym (%)**	1	0.49	0.98	0.54	0.66	0.67	0.69	0.63	1.53	0.43	1.18	0.67	0.33	0.5	0
**CRP (mg/dl)**	1.47 ± 2.35	1.66 ± 2.01	2.1 ± 3.33	2.59 ± 4.11	4.65 ± 4.69	3.19 ± 4.47	2.39 ± 3.33	1.37 ± 1.46	2.29 ± 2.06	2.07 ± 3.51	2.03 ± 2.06	1.19 ± 2.17	2.81 ± 3.46	1.985 ± 1.64	1.13 ± 1.07

Value is presented with mean ± standard deviation; Abbreviations: ADV: adenovirus; ANC: absolute neutrophil count; Boca: human bocavirus; BW: body weight; CRP: C-reactive protein; EV: enterovirus; Flu: influenza virus; Hb: hemoglobin; Hct: hematocrit; ICU: intensive care unit; Lym: lymphocyte; MPV: human metapneumovirus; PCR: polymerase chain reaction; PIV: parainfluenza virus; Plt: platelet count; RSV: respiratory syncytial virus; RV: human rhinovirus; WBC: white blood cell count.
